# Impact of Vigorous-Intensity Physical Activity on Body Composition Parameters, Lipid Profile Markers, and Irisin Levels in Adolescents: A Cross-Sectional Study

**DOI:** 10.3390/nu12030742

**Published:** 2020-03-11

**Authors:** Catia Morelli, Ennio Avolio, Angelo Galluccio, Giovanna Caparello, Emanuele Manes, Simona Ferraro, Daniela De Rose, Marta Santoro, Ines Barone, Stefania Catalano, Sebastiano Andò, Diego Sisci, Cinzia Giordano, Daniela Bonofiglio

**Affiliations:** 1 Department of Pharmacy, Health and Nutritional Sciences, University of Calabria, Rende, 87036 Cosenza, Italy; catia.morelli@unical.it (C.M.); daniela.derose89@gmail.com (D.D.R.); ms83.santoro@libero.it (M.S.); ines.barone@unical.it (I.B.); stefcatalano@libero.it (S.C.); sebastiano.ando@unical.it (S.A.); 2 Centro Sanitario, University of Calabria, 87036 Arcavacata di Rende, CS, Italy; 3 Healthy Center srl, 87100 Cosenza, Italy; ennioavolio@libero.it (E.A.); angelo.galluccio@yahoo.it (A.G.); caparello.giovanna@gmail.com (G.C.); emanuelemanes85@gmail.com (E.M.); ferrarosimona@hotmail.it (S.F.)

**Keywords:** physical activity, body composition, Mediterranean diet, irisin, lipid profile marker, healthy lifestyle

## Abstract

In adolescence, health status is influenced by several factors, including dietary pattern and physical activity (PA) which are crucial elements of lifestyle in terms of prevention and treatment of metabolic and chronic diseases. The current study aimed to explore the impact of the different intensity levels of PA along with the adherence to a Mediterranean diet (MD), on body composition indices and metabolic parameters in a cohort of adolescents, thereby investigating potential predictors of health behavior in youth. This cross-sectional study was carried out among 92 participants (44 girls and 48 boys, aged 14 to 17 years), which were divided into the following three groups according to intensity levels of PA: Group A (physical inactivity), Group B (moderate PA), and Group C (vigorous-intensity PA). The Questionnaire of Adherence to the Mediterranean Diet (KIDMED test) was used to assess both diet composition and adherence to a MD. All subjects underwent anthropometric measurements, bio-impedentiometric analysis for body composition parameters, and biochemical and hormonal measurements. The majority of adolescents (60.87%) had a medium adherence to the MD, and even a better distribution of food rates was found in adolescents performing vigorous-intensity PA. A comparison of anthropometric measurements and body composition parameters among groups showed that body mass index and fat mass (FM) were significantly lower while body cell mass (BCM), free fat mass (FFM), phase angle (PhA), and total body water (TBW) were higher in Group C adolescents as compared with those of Group A. In Group C, insulin resistance (HOMA-IR) was reduced and insulin levels were inversely associated with FFM (*r* = −0.454 and *p* = 0.004) and directly correlated with FM (*r* = 0.331 and *p* = 0.003). In the same Group C, we observed elevated serum irisin levels and lower lipid profile markers as compared with Group A. Interestingly, irisin negatively correlated with both total cholesterol (*r* = −0.428 and *p* = 0.04) and LDL (*r* = −0.468 and *p* = 0.02) in Group C. Finally, a receiver operator characteristic curve (ROC) analysis revealed irisin, LDL, HDL, and body composition variables (FFM, BMC, PhA, and TBW) as the most predictive measures for vigorous-intensity PA. Our results highlight the importance of developing healthy lifestyle programs that include improving the intensity of PA among a young population as a superior strategy for ensuring a better quality of life.

## 1. Introduction

Adolescence is the period of development that begins at puberty and ends at adulthood and is characterized by rapid physiological changes in which adequate nutrition is crucial for achieving full growth potential. Given the close intertwining between nutrition and the adolescent transition, energy and nutrition requirements must match the needs of the adolescents as they typically engage in physical work or recreational exercise which benefit the strengthening of muscle mass. In addition, adolescence food intake is also a significant predictor of nutritional habits in adulthood [1]. In the last decades, globalization has led to a rise in the so-called “Western” dietary pattern, characterized by the presence of foods with high quantities of refined carbohydrates, sugars, salt, saturated fats, proteins, as well as quantities of fruits and vegetables [2,3]. This phenomenon has created a “nutritional transition”, where problems such as being overweight, obesity, and diet-related chronic diseases have become new challenges for public health systems in Mediterranean countries [4]. In contrast to this trend and in the context of overall dietary habits, the Mediterranean diet (MD) has been accepted as one of the healthiest dietary patterns in the world [5], showing significant protection against the most common chronic and metabolic diseases [6,7,8]. In this dietary pattern, the consumption of cereals, legumes, nuts, fish, and olive oil predominates and there is a low intake of red meat and processed foods. Although there are different procedures to evaluate the level of adherence to the MD, the Questionnaire of Adherence to the Mediterranean Diet (KIDMED test) has been extensively used as an effective tool to assess the quality of food habits and, specifically, to determine the degree of adherence to the MD in children and young people [9]. Moreover, a healthy MD lifestyle habits also include an adequate level of physical activity [2,3].

Physical activity (PA), defined as ”any bodily movement produced by skeletal muscles that results in energy expenditure” [10,11], is a vital part of a healthy lifestyle with beneficial effects in children and adolescents. Some of the benefits in a population include reductions in blood cholesterol, hypertension, metabolic syndrome, obesity, and associated metabolic and chronic illnesses such as diabetes mellitus type 2 and cardiovascular diseases [12,13,14,15,16,17,18]. Several PA tracking studies have analyzed activity patterns in childhood and adolescence and the risk of maintaining sedentary behaviors that can induce morbidity [19]. A low level of PA is associated with metabolic risk factors in young people that can also persist until adulthood [20]. The benefits of PA, exercise, and sports are mediated by metabolic and molecular remodeling of skeletal muscle [21], and also by the release of cytokines from muscle, termed myokines [22]. Among the biologically active substances secreted by myocytes, irisin, discovered by Boström et al. in the last decade, is a pleiotropic hormone mediating the beneficial effects of PA such as augmented energy expenditure or fat oxidation [23]. In human studies, exercise has been shown to significantly increase irisin concentrations [23,24,25]. Particularly, levels of irisin peak after 3 to 60 min of exercise and return to baseline 6 h later [26]. The type of acute exercise can also affect irisin, with some studies suggesting that aerobic exercise, as well as other resistance exercises or heavy strength training, stimulates the increase in circulating levels of irisin [26,27,28]. Some authors have reported that chronic exercise did not alter circulating levels of irisin [29,30], however, up to now, the impact of PA intensity levels on irisin concentration in the blood is not completely clarified. Therefore, the aim of this study was to explore the impact of the different intensity levels of PA, as well as the adherence to a MD, on body composition indices, metabolic parameters, and irisin in a cohort of adolescents, thereby investigating potential predictors of a health status in young population.

## 2. Materials and Methods

The present research was a cross-sectional, descriptive, and quantitative study aimed at evaluating different intensity levels of PA and adherence to the MD on anthropometric indices, body composition parameters, and biochemical and hormonal measurements in adolescents enrolled in the trial Di, Me, Nu (Dieta Mediterranea e Nuoto-Mediterranean Diet and Swimming) founded by the EU Regional Operational Programme Calabria, Italy (POR Calabria FESR-FSE 2014–2020) (prot. #52243/2017). All participants and their parents received a detailed explanation of the study’s purposes. Thus, we obtained a verbal consent from all the enrolled adolescents, after they were informed that participation was voluntary. Parents of adolescents were provided written informed consent prior to the adolescents’ enrolment in the trial. This study was conducted according to the guidelines laid down in the Declaration of Helsinki and approved by the Ethic Committee of the University of Calabria, Italy (#5727/2018).

### 2.1. Study Population

A total of 92 subjects, including 44 girls and 48 boys, aged 14 to 17 years, were recruited from students of the public high school “Istituto Istruzione Superiore”-Castrolibero (CS) and several swim, soccer, and squash teams located in Calabria Region, Italy, during the period from December 2018 to January 2019. The exclusion criteria included lack of completing the questionnaire of adherence to a MD, evident health-related problems, use of medications, any kind of restrictive diet (i.e., hypocaloric, low carbohydrate, and low fat). Participants did not have any kind of cognitive or physical/motor limitation.

### 2.2. Nutritional History Assessment

The nutritional and medical history of participants were investigated during an oral interview by a team of professionals in nutrition, using a nutritional history record, as previously reported [31,32]. Our interview included the following sections: general data (date of birth and sex); medical history (illness, taking medications, vitamins, minerals or other dietary supplements, presence of allergy and food allergy, and food intolerance); nutritional habits to assess usual food intake and eating patterns; physical activity habits (Supplementary Table S1). On the basis of the WHO recommendations [33], the intensity of physical activity (PA) levels were classified as follows: physical inactivity (<3 metabolic equivalents (METs), moderate PA (3 to 6 METs), and vigorous-intensity PA (>6 METs). On the basis of at least 60 min daily of moderate-intensity PA, such as bicycling, dancing, brisk walking, gymnastics, ballet, aquatic aerobics, recreational swimming or vigorous-intensity PA such as jogging or running, boxing, tennis, soccer, basketball, squash, swimming, aerobic dancing, and volleyball the enrolled population was divided into the following three groups: Group A (physical inactivity, 25 subjects), Group B (moderate-intensity PA, 35 subjects) and Group C (vigorous-intensity PA, 32 subjects).

### 2.3. Questionnaire of Adherence to the Mediterranean Diet (KIDMED Test)

The KIDMED test, used to assess the adherence to the MD in the study population [9], was updated according to the new MD pyramid by the International Foundation of Mediterranean Diet (IFMed) [34]. The MD adherence score, from 0 to 12, was based on a 16-point test that was administered through a paper questionnaire (Supplementary Table S2). Questions denoting a positive aspect with respect to the MD were assigned a value of +1 (consumption of fruits, vegetables, fish, legumes, whole cereals or grain, nuts, oil, dairy products, and yogurt) and those with a negative connotation −1 (skipping breakfast, consumption of baked goods, sweets, and going to a fast food). The sum of the values from the questions was classified into the following three levels of MD adherence: optimal (≥8 points), medium (4 to 7 points), and poor (≤3 points) [9].

### 2.4. Anthropometric Parameters

The basic anthropometric data were collected using validated protocol by the anthropometric evaluation manual of nutritional status [35]. Participants’ weight was determined using KERN MPC 250K100M with a capacity of 250 kg and a precision of 100 g. Height was determined using a Seca stadiometer (Model 206, Seca Deutschland, Hamburg, Germany), which has a maximum capacity of 220 cm and precision of 1 mm. Weight and height were measured with subjects wearing light clothing and no shoes. Body circumferences (waist and hip) of each participant were measured by Seca 201 validated ergonomic circumferences measuring tape, with a measurement range of 1 to 205 cm and a division of 1 mm. Weight and height were used to calculate the body mass index (BMI), i.e., weight in kilograms divided by height in meters squared.

### 2.5. Bioelectrical Impedance Analysis

Body composition evaluation was performed after a 12 h overnight of fasting. Each subject underwent a bio-impedentiometric analysis (BIA) (single-frequency 50 kHz BIA 101 S, Akern/RJL Systems, Florence, Italy) which was carried out to evaluate resistance, reactance, phase angle (PhA), total body water (TBW), body cell mass (BCM), free fat mass (FFM), and fat mass (FM). The analysis was carried out with tetrapolar electrode placement and subjects were in a supine position. Shoes and socks were removed, and contact areas were scrubbed with alcohol immediately before electrode placement. Electrodes (BIATRODES Akern Srl Florence, Italy) were placed proximal to the phalangeal metacarpal joint on the dorsal surface of the right hand and distal to the transverse arch on the superior surface of the right foot. Sensor electrodes were placed at the midpoint between the distal prominence of the radius and ulna of the right wrist, and between the medial and lateral malleoli of the right ankle according to measurement procedure protocol [36]. Data were analyzed by Bodygram Plus (software) Version 1.2.2.8. (Akern Srl; Florence, Italy).

### 2.6. Biochemical Measurements

Venous blood samples were collected after 8 to 10 h overnight fasting conditions. Serum was obtained after centrifugation at 3000 rpm for 10 min and stored in sterile tubes at 4 °C for no longer than 4 h during the morning of collection. Biochemical parameters were determined on a Konelab 20i Chemistry Analyzer (Thermo Electron Corporation, Vantaa, Finland) according to the standardized procedures. Afterward, the serum samples were stored at −80 °C.

### 2.7. Hormonal Level Assessments

Serum insulin levels were measured with an Enzyme-Linked Immunosorbent Assay (ELISA) kit (Novatec Immundiagnostica GmbH, Dietzenbach, Germany) following the manufacturer instructions. The lowest detectable concentration of insulin was 0.25 µIU/mL at a 95% confidence limit, the intra-assay variability was within ≤5%. Insulin action was expressed as HomeOstasis Model Assessment for estimating Insulin Resistance (HOMA-IR) which was calculated as the product of fasting glucose concentration (mg/dL) and fasting insulin concentration divided by 405. Serum irisin concentration was measured with an ELISA kit (Cell Biolabs Inc, San Diego, CA, USA) as recommended by the manufacturer. The kit has a detection sensitivity limit of 6.25 ng/mL irisin.

### 2.8. Statistical Analysis

Data were analyzed using SigmaPlot Version 12.0 (Systat, San Jose, CA, USA). A Kolmogorov–Smirnov test (with Lilliefors’ correction) was used to verify data normality. Data were reported as the mean and standard deviation (SD). The data that was not normally distributed were log10-transformed before evaluating statistical differences between samples by using parametric tests (one-way ANOVA and Student’s *t*-test). Linear regression was used to evaluate the relationship between independent and dependent variables. The regression coefficient was used to graph the straight line that most closely described the association between variables, and the statistical significance was evaluated by Pearson’s correlation test. Qualitative variables were described as frequencies (%) with respect to an ideal situation (100% compliance) and the statistical differences were evaluated by Chi-squared tests and summarized in graph (radar plot) and tables. The overall accuracy of each predictor of physical activity (irisin, LDL, HDL, BMI, FFM (%), BCM (kg), PhA and TBW) was determined by a receiver operating characteristic (ROC) curve analysis by using MedCalc for Windows, v 8.1 (MedCalc Software, Ostend, Belgium). The area under the ROC curve (AUC) was drawn to determine the accuracy with a 95% confidence interval (CI) expressing the sensitivity and specificity of each predictor of physical activity. AUC ≥ 0.5 was chosen to discriminate the marker having a predictor value of physical activity, and the optimal cutoff points for each predictor factor was determined according to Youden index maximum points. Statistical significance was set at *p* < 0.05.

## 3. Results

Anthropometric characteristics, KIDMED score, and physical activity levels of participants enrolled within the Italian project Di, Me, Nu are presented in Table 1. The mean age of the total population studied was 15.76 (±0.99) years without gender differences. The MD adherence evaluated by the KIDMED score was 5.86 (±2.41) for the total adolescent sample independent of sex, indicating a medium adherence to the MD (Table 1).

**Figure 1:**
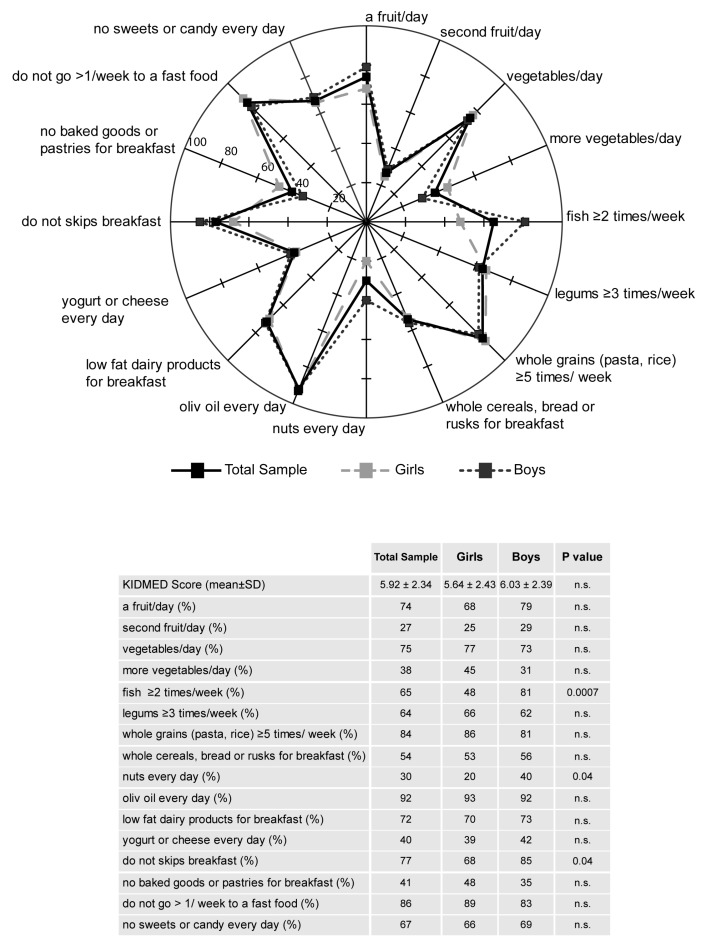
Compliance with items from the KIDMED test in the total sample, girls and boys. The radar chart plots the values of each item of Mediterranean diet score along a separate axis that starts in the center of the chart (0% compliance) and ends at the outer ring (100% compliance). The KIDMED score (M ± SD) and frequency (%) of the population adherent to each recommendation are reported in the table. Statistical differences were evaluated by Chi-squared tests. *p* < 0.05, girls vs. boys.

On the basis of the KIDMED test, the compliance rates for each food were calculated and depicted in radar charts, which illustrate the gap between the current state (percentage of participants currently adhering to each dietary recommendation) and the ideal situation (100% compliance) in the total population sample, as well as in girls and boys (Figure 1). Importantly, in the entire population, the intake of a second fruit (27%), nuts (30%), more vegetables (38%), and yogurts or cheese (40%) resulted definitively outside the recommendations, furthermore, those who consumed baked goods or pastries for breakfast (59%) were also located outside current nutritional guidelines (Figure 1). A comparison of girls and boys showed significant differences for the intakes of fish (48% vs. 81%, *p* < 0.001) and nuts (20% vs. 40%, *p* < 0.05) and for the habit of having breakfast (68% vs. 85%, *p* < 0.05), respectively (Figure 1).

In Table 1, based on the different levels of the intensity of physical activities or exercise established by the WHO [33], we classified the whole sample into the following three groups: Group A of physical inactivity in which adolescents did not achieve physical activity guidelines [11], Groups B and C categorized according to their moderate- and vigorous-intensity PA, respectively. According to this categorization, body composition parameters were evaluated in all subjects enrolled and in relation to gender, which were analyzed separately (Table 2). Specifically, significant differences were observed in the total sample for all the variables (BMI, PhA, BCM, FFM, FM, and TBW) as comparing with Group A vs. Group C. No significant differences in the majority of body composition parameters were found between Group A and Group B, with the exception of the BMI and FM (expressed in kg) which was lower in Group B as compared with Group A (21.93 ± 2.17 vs. 24.56 ± 5.41, *p* < 0.01 and 12.80 ± 5.67 vs. 18.09 ± 9.99, *p* < 0.05, respectively), and BCM (expressed in kg) and FFM (expressed in percentage) was higher in Group B as compared with Group A (32.19 ± 5.32 vs. 31.95 ± 5.08, *p* < 0.01 and 78.62 ± 8.88 vs. 73.74 ± 10.61, *p* < 0.05, respectively) (Table 2). When analyzing body composition parameters by gender, in girls, we did not observe any significant variation among the three different PA groups, with the exception of the BMI which was lower in Group C vs. Groups A and B (20.65 ± 1.73 vs. 23.18 ± 2.42 and 22.02 ± 2.25, *p* < 0.01, respectively). Similarly, BCM (in kilograms) was lower in Group C vs. Groups A and B (28.45 ± 3.46 vs. 32.18 ± 5.32 and 29.91 ± 4.21, *p* < 0.05, respectively), while TBW resulted higher in Group C vs. Groups A and B (60.1 ± 4.73 vs. 53.61 ± 7.06 and 54.34 ± 4.66, *p* < 0.05, respectively). In boys, statistically significant differences for all the variables analyzed were found (Table 2).

**Figure 2:**
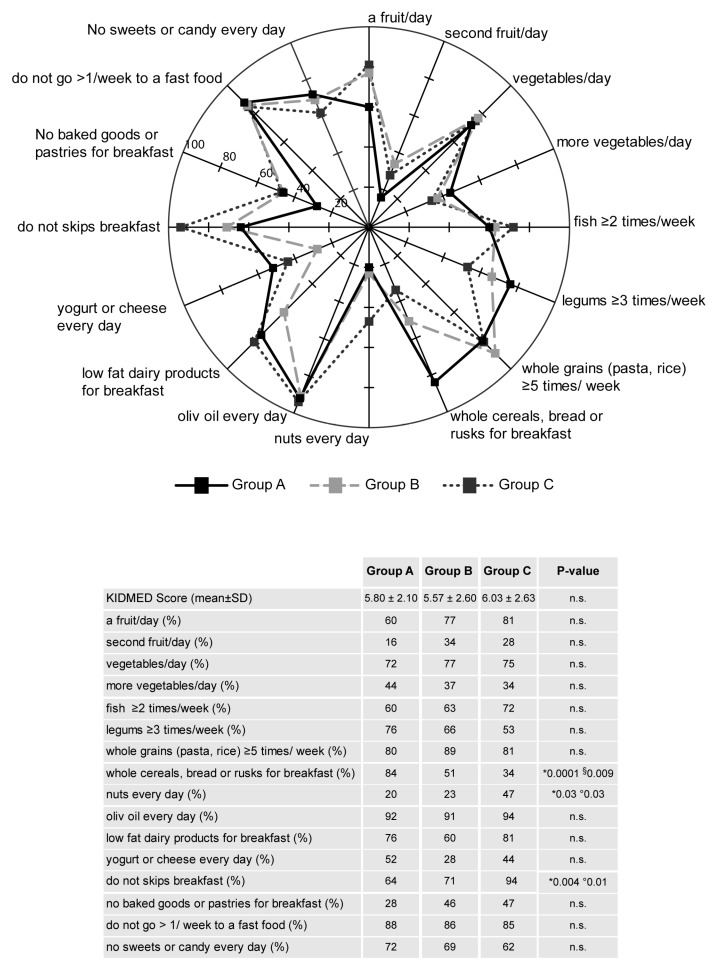
Compliance with items from the KIDMED test in the three physical activity groups (Group A, B and C). The radar chart plots the values of each item of Mediterranean diet score along a separate axis that starts in the center of the chart (0% compliance) and ends at the outer ring (100% compliance). The KIDMED score (M ± SD) and frequency (%) of the population adherence to each recommendation are reported in the table. Statistical differences were evaluated by Chi-squared tests. *p* < 0.05, * Group A vs. Group C; ° Group B vs. Group C; § Group A vs. Group B.

Analyzing population sample into the three intensity levels of PA, no differences in the KIDMED score were found (Figure 2). However, in the three groups, the differences in the compliance rates for each food were also calculated and depicted in radar charts, as shown in Figure 2. Although among the three groups of PA levels there were no significant differences in the food rates for most of the dietary recommendations, adolescents of Group C showed the intake of nuts higher than those of Groups B and A (47% vs. 23% and 20%, *p* < 0.05), as well as the percentage of subjects having breakfast was higher in Group C as compared with Groups B and A (94% vs. 71% and 64%, *p* < 0.05), even the intakes of whole cereals, bread, and rusks for breakfast were 34% vs. 51% and 84% in Group C vs. Groups B and A, respectively (*p* < 0.05) (Figure 2).

**Figure 3:**
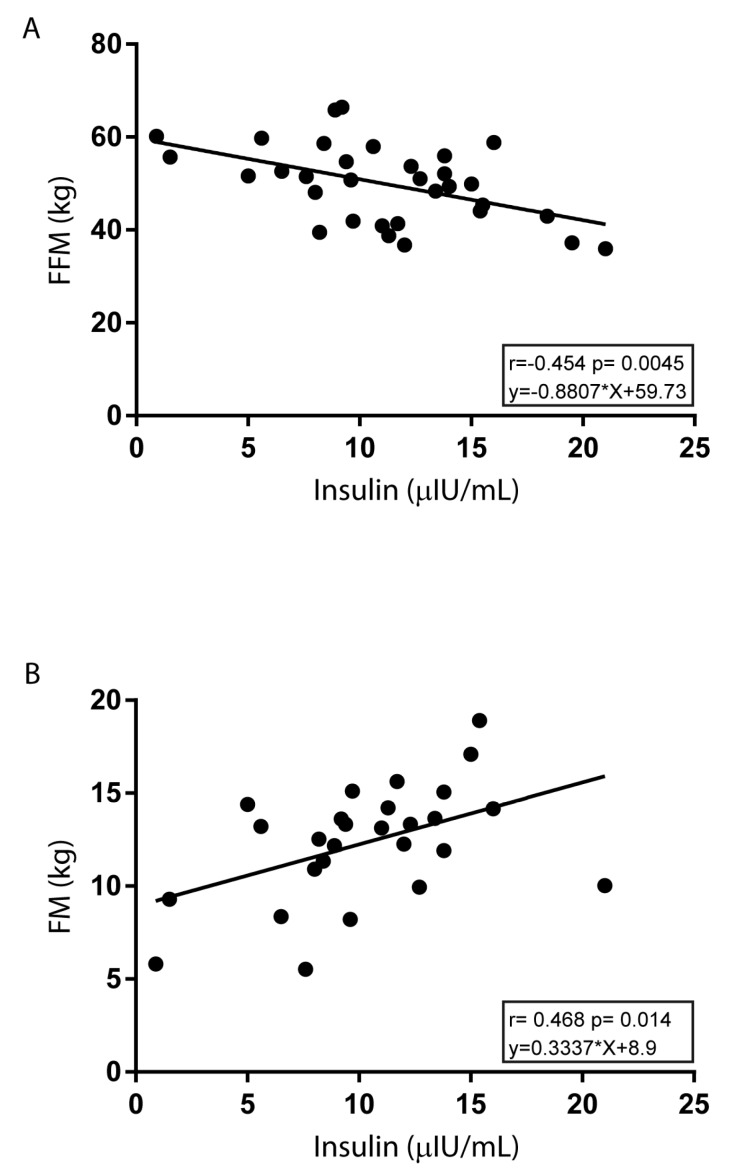
Correlations between insulin and FFM (kg) or FM (kg) in Group C. Association of insulin with FFM (kg) (**A**) or FM (kg) (**B**) were analyzed by Pearson’s correlation test. For each linear regression graph, the linear equation (y), the correlation coefficient (*r*), and the statistical significance (p) are reported.

Table 3 shows the metabolic profile results for all subjects and in relation to gender according to different levels of PA intensity. We found, in the total sample, a significant increase in serum glucose levels as comparing with Group C vs. Group A (85.85 ± 6.37 vs. 81.72 ± 7.05, *p* < 0.05) and for the presence of reduced serum insulin levels between Groups C and B vs. Group A (9.74 ± 3.78 and 9.13 ± 4.20 vs. 12.61 ± 5.97, *p* < 0.05). To be noted, in adolescents from Group A, HOMA-IR values were significantly higher as compared with those from Group B (2.56 ± 1.22 vs. 1.84 ± 0.85, *p* < 0.05) and a similar reduced trend was found in Group C. When analyzing differences in these metabolic parameters for gender, we observed a significant reduction in insulin levels in boys of Group C vs. Group A (9.54 ± 4.14 vs. 13.93 ± 7.94, *p* < 0.05), as well as in HOMA-IR values (2.06 ± 0.91 vs. 2.89 ± 1.61, *p* < 0.05). In addition, in the total sample of Group C, insulin was inversely associated with FFM (r = −0.454, *p* = 0.004) and directly associated with FM (r = 0.331 and *p* = 0.003) (Figure 3). Concerning other biochemical parameters, it was interesting to note that LDL levels were significantly lower in the total sample, as well as in girls of Group C than those in Group A (75.91 ± 20.97 vs. 97.91 ± 29.42, *p* < 0.05 total sample; 71.12 ± 23.48 vs. 108.08 ± 26.63, *p* < 0.05 girls), conversely HDL levels were significantly higher in the total sample, as well as in boys of Group C as compared with those in Group A (63.47 ± 12.44 vs. 53.80 ± 12.52, *p* < 0.05 total sample; 61.33 ± 11.41 vs. 53.08 ± 13.52, *p* < 0.05 boys). Moreover, statistically significant differences were observed among groups with higher levels in Group C vs. Groups A and B for urea in total sample (33.09 ± 5.88 vs. 27.48 ± 6.72 and 28.54 ± 7.00, *p* < 0.01), whereas in girls, higher levels were observed in Group C vs. Group A (30.00 ± 4.50 vs. 24.30 ± 5.51, *p* < 0.05). More interestingly, total adolescents from Group C, as well as girls, had significantly higher irisin levels than those from Group A (55.02 ± 81.19 vs. 12.3 ± 8.88, *p* < 0.05, total sample; 27.05 ± 16.14 vs. 12.93 ± 8.92, *p* < 0.05, girls) (Table 3).

**Figure 4:**
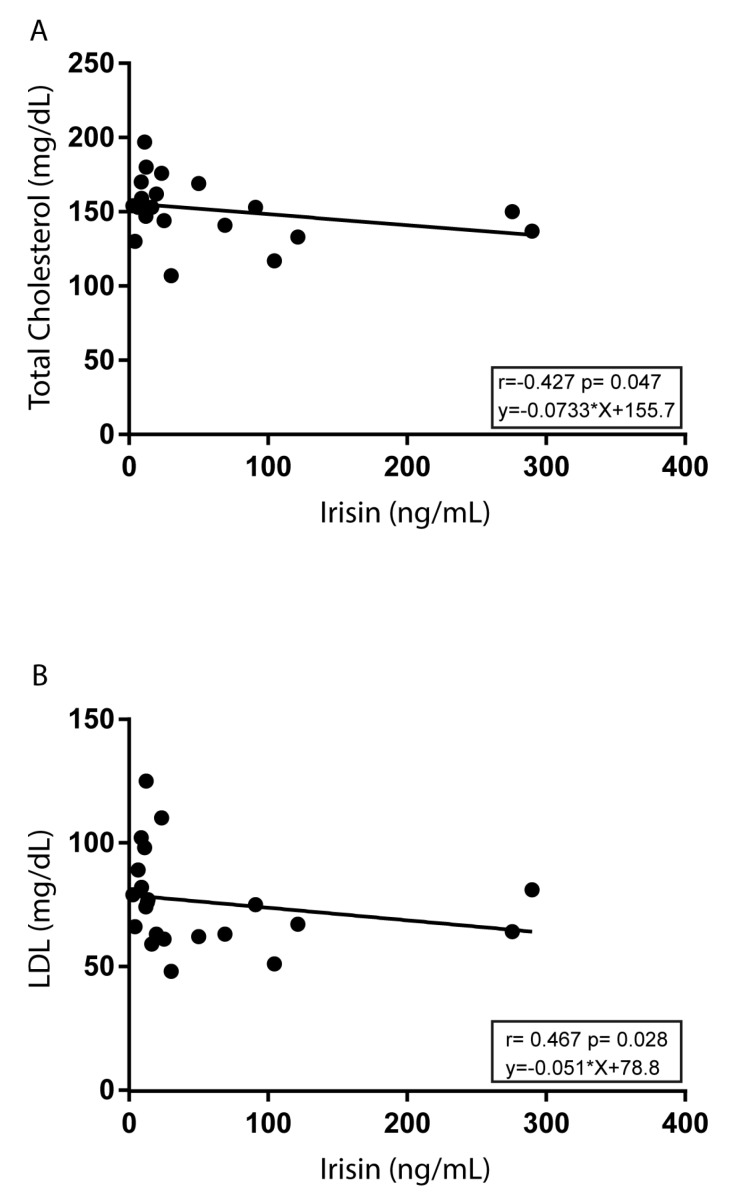
Correlations of irisin with total cholesterol and LDL in Group C. Association of irisin with total cholesterol (**A**) or LDL (**B**) were analyzed by Pearson’s correlation test. For each linear regression graph, the linear equation (y), the correlation coefficient (*r*), and the statistical significance (p) are reported.

Interestingly, assessing the correlation of irisin with metabolic parameters, we found a negative association between irisin and both total cholesterol (*r* = −0.428 and *p* = 0.04) and LDL (*r* = −0.468 and *p* = 0.02) in Group C (Figure 4).

Finally, we analyzed the usefulness of anthropometric indices, body composition parameters, lipid profiles, and irisin as predictors of vigorous-intensity PA with respect to inactivity in adolescents (Group C vs. Group A). Table 4 shows AUC, optimal cutoffs, and measures of accuracy for irisin, LDL, and HDL values (AUC > 0.7), for BMI (AUC > 0.6) and selective body composition variables (AUC > 0.7). Particularly, FFM and TBW had the highest sensitivity (>90%), while irisin, BMC, and PhA showed the highest specificity (>80%).

**Figure 5:**
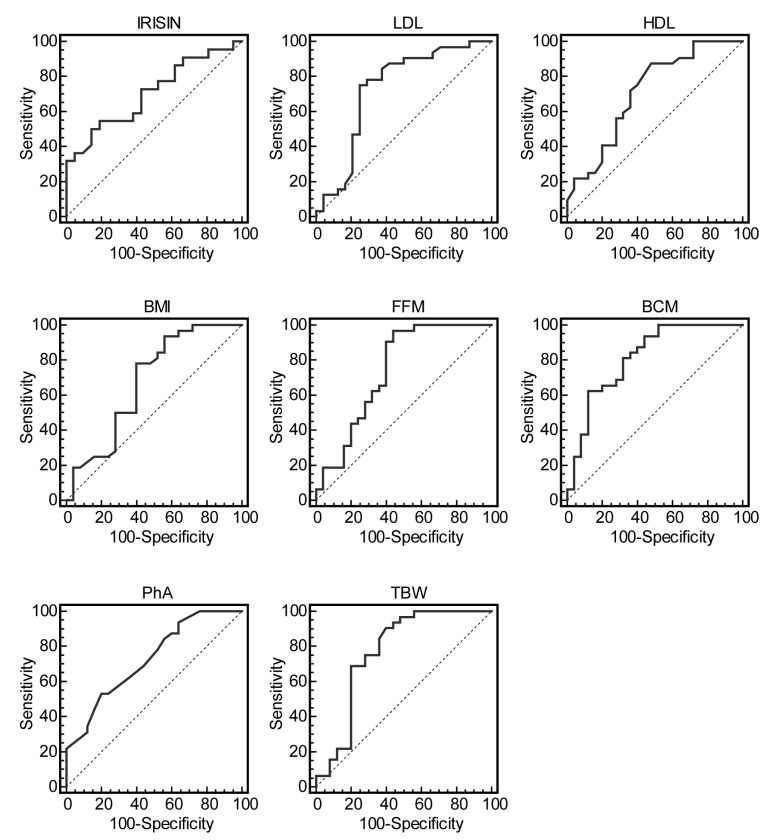
Receiver operating characteristic (ROC) curves of irisin, LDL, HDL, BMI, FFM, BCM, PhA, and TBW as predictors of vigorous-intensity physical activity. Graphic evaluation of irisin, LDL, HDL, BMI, FFM, BCM, PhA, and TBW with respect to physical activity (Group C vs. Group A), showing the true-positive rate (sensitivity) and the false-positive rate (specificity) of the analysis as a function of all possible cut-points for the analyzed markers. The analysis was performed with ROC curves, as described in the Statistical Analysis Section.

The ROC curves for the most predictive measures are shown in Figure 5.

## 4. Discussion

The present study aimed to analyze and describe the impact of PA and adherence to a MD on body composition, as well as on health-related metabolic and hormonal parameters in Italian adolescents. The main findings are that adolescents performing vigorous-intensity PA have the following characteristics: (1) better distribution of compliance with food-related recommendation of MD, (2) body composition parameters reflecting lean metabolic active body mass, (3) reduced insulin concentrations and insulin resistance, (4) lower lipid profile markers, and (5) elevated serum irisin levels.

It is widely accepted that adherence to the MD pattern has been shown to be associated with a better health status, due to its protective effects against a wide range of chronic diseases, including obesity, type 2 diabetes mellitus, metabolic syndrome, cardiovascular diseases, dementia, and several cancers [5,6,37,38,39]. Recently, higher MD adherence was found to be positively correlated to well-being in a young population [40]. Thus, it is worth highlighting the importance of promoting the MD model, particularly during adolescence as a tool to achieve an overall healthy condition that improves life expectancy. In this cross-sectional study, the results of the KIDMED test (poor 16.3%, medium 60.87%, and optimal adherence 22.83%) were similar to those of studies carried out in other countries in which a medium adherence to the MD was reported [41,42,43,44,45]. Furthermore, no significant differences were found in adherence to the MD according to gender, in agreement with the data reported by other authors among adolescents living in the Mediterranean region [42]. We have also recently found an average adherence to the MD in the adult population from the same Mediterranean area, which was independent of sex, but directly associated with age [46]. Importantly, in all samples, the percentage of adherers to recommendations for fruits, nuts, and fish, estimated by a validated 14-point Mediterranean adherence screener (MEDAS) result was outside the dietary guidelines [46]. Referring to the evaluation using the KIDMED to assess the adherence to the MD in our adolescent sample, the compliance rates for consuming yogurts or cheese every day, more vegetables a day, nuts every day, and a second fruit each day resulted in being definitively outside the recommendations. When analyzing the results of dietary habits by gender, surprisingly boys were more compliant in fish and nuts intake and had less tendency to skip breakfast. However, this research underlines the need to reinforce the adherence to the MD in adolescents, since emerging experimental evidences have been recurrently established that a MD, rich in fiber, minerals, vitamins, omega-3 fatty acids, and monounsaturated fatty acids, provides an equilibrated mix of nutrients with beneficial effects due to their antioxidant, anti-inflammatory, probiotic and antineoplastic functions [47].

More importantly, in association with adherence to a MD, PA is a crucial indicator of healthy lifestyles, since it is considered to be a cornerstone in the prevention and treatment of chronic diseases and age-related muscle wasting [48,49]. The benefits of PA or better exercise or sport are related to an adequate body composition that, in turn, can assure a good physical fitness and/or high rate of adherence to a MD along with high levels of a health-related quality of life [50,51]. Interestingly, when categorizing our population sample of adolescents according to physical inactivity or different intensity levels of PA, we found in Group C a better compliance in nuts intake and a lower tendency to skip breakfast. It is worthwhile to highlight that, although the MD adherence was similar among adolescents studied, subjects performing vigorous-intensity activity showed an overall healthier diet. In addition, profound differences in anthropometric measurements and body composition parameters assessed by the BIA method among groups were observed. Particularly, the comparison between inactive subjects and those engaged in regular vigorous PA displayed reduced BMI values concomitant with decreased FM, while BCM indices and the percentages of TBW were significantly increased. Of note, the PhA values, that reflect body cell mass, the integrity of the cell wall, cellular health, and, consequently, the health of the individual [52] were higher in adolescents grouped according to their vigorous-intensity PA as compared with inactive subjects. Our results are in line with data from a recent meta-analysis showing that PA had a positive effect on PhA that were significantly higher in the active group than in the control group, suggesting the importance of routinely including exercise in health care [53]. When analyzing the data by gender, significant differences were observed for anthropometric and all body composition variables tested between boys of Group C as compared with Group A, while in girls significant differences were observed for BMI, BCM expressed in kilograms, and TBW, likely due to the diverse adaptation, also mediated by hormonal status, to the same level of PA.

Beneficial effects of PA are mediated by metabolic and molecular remodeling of skeletal muscle able to modulate glycemic and lipid profiles [21]. Indeed, adolescents performing vigorous-intensity PA had increased levels in fasting glycemia, along with a significant reduction in insulin levels as compared with their inactive counterparts, that conversely had higher values of HOMA-IR, reflecting a higher insulin resistance in all samples and in boys. These results endorse that physical exercise activates the insulin-signaling pathways and facilitates the process of glucose diffusion via GLUT-4 [54]. Thus, sensitivity to insulin action is increased and lower levels of insulin release are required for glucose uptake. Furthermore, insulin was also inversely correlated to FFM in adolescents of Group C, clearly underlining the impact of this hormonal parameter on body composition in relation to the level of PA. Controversial data has been reported regarding the association between PA and lipid profile in a young population [55,56,57,58]. In agreement with previous studies [59,60], we found a lipid profile marker which consisted of lower LDL levels and higher HDL concentrations in Group C as compared with Group A, whereas TG levels were unmodified among groups.

The favorable effects of PA could be, at least in part, achieved by so-called exercise mimetics, including cytokines released from muscle and termed myokines, which communicate with other tissues and act locally on muscle to exert metabolic effects in an autocrine/paracrine/endocrine manner [22]. Among myokines, irisin has been suggested to mediate some of the beneficial effects of PA by inducing uncoupling protein 1 and, subsequently, increasing energy expenditure in white adipocytes [23]. However, currently, there is growing interest in exploring the impact of different levels of PA intensities in circulating serum irisin. In our study, according to the PA intensity levels, we observed a trend toward a progressive increase in irisin concentration, which showed the highest levels in adolescents engaged in regularly vigorous PA. More importantly, in the same subjects, a negative association between irisin and both total cholesterol and LDL levels was observed, suggesting irisin as a potential favorable link between PA and lipid metabolism. Analyzing the usefulness of irisin, as well as the lipid profile, as predictors of vigorous-intensity PA in adolescents using AUC of ROC curves, we observed an appreciable accuracy of these predictors. Similarly, results obtained from ROC analysis for FFM, BCM, PhA, and TBW suggest that these selective body composition variables are predictable markers of PA.

There are a number of potential limitations of the study that need to be taken into account when interpreting the results. First, this is an observational and cross-sectional investigation, limiting the ability to make causal inferences about the association between irisin and body composition, as well as lipid profile in relation to PA. Another limitation of the study was the relatively small sample size, particularly for some PA groups (e.g., girls of Group C) that could potentially impact on the statistically differences of the results when evaluated by gender, and a suspected confounding effect of hormonal changes occurring during puberty. However, results from the present study document profound differences in anthropometric, body composition, biochemical, and hormonal parameters, as well as in food-related recommendation of MD in adolescents categorized into different intensity PA level groups. Future studies with more potential factors, larger number of participants, and using objective methods to measure physical activity are needed to confirm our results.

## 5. Conclusions

In summary, we found that serum irisin levels were higher in active than in inactive adolescents and negatively correlated with unhealthy metabolic parameters in subjects performing vigorous-intensity PA. Our findings highlight the importance of developing prevention programs that include improving the intensity of PA, and, in addition, adherence to a MD among adolescents, given that this combination is a superior strategy for ensuring a better quality of life.

## Figures and Tables

**Table 1:** Anthropometric characteristics, Mediterranean diet adherence (KIDMED), and physical activity levels of all participants and differentiated by gender.

Characteristics	Total (*n* = 92)	Girls (*n* = 44)	Boys (*n* = 48)
Age (years)	15.76 ± 0.99	15.82 ± 0.99	15.71 ± 1.01
Weight (Kg)	62.23 ± 12.28	56.86 ± 7.54	67.15 ± 13.72
Height (cm)	167.01 ± 12.42	162.51 ± 15.96	171.12 ± 5.43
BMI (Kg/m^2^)	22.58 ± 3.59	22.12 ± 2.34	23.01 ± 4.45
Waist (cm)	72.43 ± 8.05	69.44 ± 5.22	75.17 ± 9.18
Hip (cm)	94.74 ± 8.34	93.37 ± 7.12	94.07 ± 9.39
Waist/hip ratio	0.78 ± 0.06	0.75 ± 0.04	0.78 ± 0.04
KIDMED score	5.86 ± 2.41	5.64 ± 2.43	5.94 ± 2.52
PA			
Group A	*n* = 25	*n* = 13	*n* = 12
Group B	*n* = 35	*n* = 23	*n* = 12
Group C	*n* = 32	*n* = 8	*n* = 24

BMI, body mass index; PA, physical activity; Group A, physical inactivity; Group B, moderate-intensity PA; Group C, vigorous-intensity PA.

**Table 2:** Body composition according to three physical activity groups in all the sample and differentiated by gender.

		Group A	Group B	Group C	*p* Value
		Total *n* = 25	Total *n* = 35	Total *n* = 32	
		Girls *n* = 13	Girls *n* = 23	Girls *n* = 8	
		Boys *n* = 12	Boys *n* = 12	Boys *n* = 24	
Height (cm)	Total	165.76 ± 8.88	164.69 ± 7.64	167.39 ± 7.47	n.s.
	Girls	159.62 ± 5.96	160.78 ± 5.48	159.70 ± 8.67	n.s.
	Boys	172.42 ± 6.36	172.17 ± 5.25	169.96 ± 4.99	n.s.
Weight (kg)	Total	67.76 ± 18.19	59.49 ± 7.14	60.91 ± 9.37	n.s.
	Girls	59.31 ± 8.70	56.91 ± 6.95	52.75 ± 6.11	n.s.
	Boys	76.92 ± 21.53	64.42 ± 6.49	63.63 ± 8.72	* 0.014, ^§^ 0.021
BMI (Kg/m^2^)	Total	24.56 ± 5.41	21.93 ± 2.17	21.72 ± 2.29	* 0.001, ^§^ 0.001
	Girls	23.18 ± 2.42	22.02 ± 2.25	20.65 ± 1.73	* 0.001, ^§^ 0.001, ° 0.027
	Boys	26.05 ± 7.27	21.72 ± 2.19	22.08 ± 2.37	* 0.001, ^§^ 0.001
PhA (°)	Total	5.89 ± 0.53	6.10 ± 0.84	6.36 ± 0.53	* 0.02
	Girls	5.80 ± 0.41	5.82 ± 0.78	6.06 ± 0.50	n.s.
	Boys	5.98 ± 0.63	6.65 ± 0.66	6.46 ± 0.51	* 0.02, ^§^ 0.02
BCM (kg)	Total	31.95 ± 5.08	32.19 ± 5.32	33.79 ± 5.73	* 0.001, ^§^ 0.001
	Girls	32.18 ± 5.32	29.91 ± 4.21	28.45 ± 3.46	* 0.001, ^§^ 0.001, ° 0.027
	Boys	31.71 ± 5.03	36.56 ± 4.50	35.58 ± 5.23	* 0.001, ^§^ 0.001
BCM (%)	Total	53.07 ± 2.72	53.99 ± 4.16	55.39 ± 2.47	* 0.02
	Girls	52.61 ± 2.17	52.58 ± 4.01	53.96 ± 2.41	n.s.
	Boys	53.56 ± 3.23	56.72 ± 3.00	55.87 ± 2.35	* 0.02, ^§^ 0.02
FFM (kg)	Total	49.67 ± 11.03	46.68 ± 7.76	49.93 ± 8.35	n.s
	Girls	41.84 ± 4.01	41.82 ± 3.09	39.45 ± 2.83	n.s.
	Boys	58.16 ± 9.84	55.99 ± 4.82	53.43 ± 6.38	n.s.
FFM (%)	Total	73.74 ± 10.61	78.62 ± 8.88	82.09 ± 7.01	* 0.002, ^§^ 0.03
	Girls	69.79 ± 9.08	74.15 ± 6.84	75.15 ± 3.93	n.s.
	Boys	78.01 ± 10.83	87.19 ± 5.34	84.40 ± 6.26	* 0.02, ^§^ 0.01
FM (kg)	Total	18.09 ± 9.99	12.80 ± 5.67	10.98 ± 4.38	* 0.003, ^§^ 0.037
	Girls	17.47 ± 6.83	15.09 ± 5.08	13.30 ± 3.47	n.s.
	Boys	18.76 ± 12.88	8.42 ± 3.99	10.20 ± 4.44	* 0.006, ^§^ 0.014
FM (%)	Tota	25.46 ± 9.87	21.37 ± 8.88	17.91 ± 7.01	* 0.004
	Girls	28.67 ± 8.00	25.85 ± 6.84	24.85 ± 3.93	n.s.
	Boys	21.99 ± 10.83	12.81 ± 5.34	15.60 ± 6.26	* 0.02, ^§^ 0.01
TBW (%)	Total	55.66 ± 7.83	57.68 ± 6.35	62.55 ± 4.98	* <0.001, ° 0.003
	Girls	53.61 ± 7.06	54.34 ± 4.66	60.1 ± 4.73	* 0.03, ° 0.01
	Boys	57.88 ± 8.31	64.08 ± 3.64	63.37 ± 4.89	* 0.009, ^§^ 0.02

BMI, body mass index; PhA, phase angle; BCM, body cell mass; FFM, fat-free mass; FM, fat mass; TBW, total body water. Statistical differences were determined by one-way ANOVA, where significantly different, Student’s *t*-test was used for pairwise comparison. ^§^ Group A vs. Group B, * Group A vs. Group C; ° Group B vs. Group C.

**Table 3:** Biochemical parameters, hormonal serum levels, and Mediterranean diet adherence (KIDMED) according to three physical activity groups in all the sample and differentiated by gender.

		Group A	Group B	Group C	*p* Value
Biochemical Parameters		Total n = 25	Total n = 35	Total n = 32	
		Girls n = 13	Girls n = 23	Girls n = 8	
		Boys n = 12	Boys n = 12	Boys n = 24	
Glucose (mg/dL)	Total	81.72 ± 7.05	81.91 ± 8.41	85.85 ± 6.37	* 0.03
	Girls	79.08 ± 6.62	79.48 ± 8.31	84.20 ± 4.32	n.s.
	Boys	84.58 ± 6.59	86.58 ± 6.68	86.24 ± 6.80	n.s.
Total cholesterol (mg/dL)	Total	162.32 ± 28.60	153.60 ± 29.75	150.75 ± 23.80	n.s.
	Girls	171.69 ± 29.70	161.35 ± 29.42	155.75 ± 29.27	n.s.
	Boys	152.17 ± 24.60	138.75 ± 25.29	149.08 ± 22.16	n.s.
LDL (mg/dL)	Total	97.91 ± 29.42	81.03 ± 23.76	75.91 ± 20.97	* 0.02
	Girls	108.08 ± 26.63	85.30 ± 24.31	71.12 ± 23.48	^§^ 0.02, * 0.01
	Boys	87.75 ± 29.57	72.83 ± 21.23	76.18 ± 19.78	n.s.
HDL (mg/dL)	Total	53.80 ± 12.52	58.46 ± 15.61	63.47 ± 12.44	* 0.02
	Girls	54.46 ± 12.04	62.69 ± 15.82	69.87 ± 13.96	n.s.
	Boys	53.08 ± 13.52	50.33 ± 11.96	61.33 ± 11.41	* 0.05, ° 0.03
Triglycerides (mg/dL)	Total	60.12 ± 20.82	69.97 ± 39.01	61.90 ± 25.11	n.s.
	Girls	63.50 ± 24.29	65.82 ± 31.28	73.87 ± 27.38	n.s.
	Boys	56.75 ± 17.07	77.92 ± 51.39	57.92 ± 23.57	n.s.
Uric acid (mg/dL)	Total	4.41 ± 1.07	4.05 ± 0.93	4.46 ± 0.77	n.s.
	Girls	3.91 ± 0.84	3.65 ± 0.55	4.29 ± 1.06	n.s.
	Boys	4.95 ± 1.06	4.81 ± 1.05	4.85 ± 0.96	n.s.
Urea (mg/dL)	Total	27.48 ± 6.72	28.54 ± 7.00	33.09 ± 5.88	* 0.005, ° 0.006
	Girls	24.30 ± 5.51	25.91 ± 6.34	30.00 ± 4.50	* 0.02
	Boys	30.92 ± 6.37	33.58 ± 5.38	34.12 ± 5.99	n.s.
Creatinine (mg/dL)	Total	0.68 ± 0.07	0.69 ± 0.10	0.70 ± 0.09	n.s.
	Girls	0.67 ± 0.07	0.65 ± 0.08	0.64 ± 0.05	n.s.
	Boys	0.70 ± 0.07	0.77 ± 0.08	0.73 ± 0.08	n.s.
Total bilirubin (mg/dL)	Total	0.68 ± 0.20	0.80 ± 0.55	0.77 ± 0.32	n.s.
	Girls	0.64 ± 0.17	0.68 ± 031	0.67 ± 0.24	n.s.
	Boys	0.72 ± 0.22	1.02 ± 0.82	0.80 ± 0.34	n.s.
Direct bilirubin (mg/dL)	Total	0.23 ± 0.08	0.23 ± 0.08	0.22 ± 0.07	n.s.
	Girls	0.23 ± 0.11	0.21 ± 0.07	0.19 ± 0.05	n.s.
	Boys	0.24 ± 0.07	0.27 ± 0.09	0.23 ± 0.07	n.s.
GOT (UI/L)	Total	19.00 ± 7.09	22.57 ± 11.60	23.31 ± 7.80	n.s.
	Girls	19.08 ± 7.83	22.04 ± 13.31	18.00 ± 3.07	n.s.
	Boys	20.50 ± 3.63	23.58 ± 7.74	25.08 ± 8.12	n.s.
GPT (UI/L)	Total	17.84 ± 8.36	17.28 ± 9.73	19.18 ± 11.75	n.s.
	Girls	15.92 ± 9.19	15.30 ± 6.86	14.75 ± 8.34	n.s.
	Boys	19.91 ± 7.17	21.08 ± 13.21	21.50 ± 12.37	n.s.
**Hormonal serum levels**					
Insulin (μIU/mL)	Total	12.61 ± 5.97	9.13 ± 4.20	9.74 ± 3.78	^§^ 0.01, * 0.04
	Girls	11.29 ± 2.78	10.12 ± 4.59	10.58 ± 1.60	n.s.
	Boys	13.93 ± 7.94	6.47 ± 2.95	9.54 ± 4.14	^§^ 0.02, ° 0.03
Irisin (ng/mL)	Total	12.30 ± 8.88	29.55 ± 34.51	55.02 ± 81.19	* 0.01
	Girls	12.93 ± 8.92	27.86 ± 39.02	27.05 ± 16.14	* 0.05
	Boys	11.60 ± 9.26	18.02 ± 17.64	61.23 ± 88	n.s.
HOMA-IR	Total	2.56 ± 1.22	1.84 ± 0.85	2.08 ± 0.83	^§^ 0.01
	Girls	2.23 ± 0.55	1.99 ± 0.93	2.13 ± 0.44	n.s.
	Boys	2.89 ± 1.61	1.40 ± 0.69	2.06 ± 0.91	^§^ 0.03, ° 0.04
KIDMED score	Total	5.80 ± 2.10	5.57 ± 2.60	6.03 ± 2.63	n.s.
	Girls	5.31 ± 1.89	5.56 ± 2.76	6.37 ± 2.33	n.s.
	Boys	6.33 ± 2.27	5.58 ± 2.39	6.17 ± 2.52	n.s

LDL, low density lipoprotein; HDL, high density lipoprotein; GOT, glutamic oxaloacetic transaminase; GPT, glutamic pyruvic transaminase; HOMA-IR, HomeOstasis Model Assessment for estimating Insulin Resistance. Statistical differences were determined by one-way ANOVA, where significantly different, Student’s *t*-test was used for pairwise evaluation. § Group A vs. B; * Group A vs. C; ° Group B vs. C.

**Table 4:** Accuracy of irisin, lipid profile measures, anthropometric and body composition parameters as predictors of the intensity level of physical activity.

Characteristics	AUC	Optimal Cutoff	Sensitivity (%) (95% C.I.)	Specificity (%) (95% C.I.)	*p* Value
Irisin (ng/mL)	0.705	>17.6	50 (28.2–71.8)	85.7 (63.6–96.8)	0.01
LDL (mg/dL)	0.729	≤82	75 (56.6–88.5)	75 (53.3–90.2)	0.001
HDL (mg/dL)	0.711	>51	87.5 (71.0–96.4)	52 (31.3–72.2)	0.002
BMI (Kg/m^2^)	0.676	≤23.5	78.1 (60.0–90.7)	60 (38.7–78.8)	0.01
FFM (%)	0.740	>71	96.9 (83.7–99.5)	56 (34.9–75.6)	0.0002
BCM (Kg)	0.814	>33.6	62.5 (43.7–78.9)	88 (68.8–97.3)	<0.0001
PhA (°)	0.727	>6.3	53.1 (34.8–70.9)	80 (59.3–93.1)	0.0006
TBW (%)	0.770	>57.4	90.6 (75.0–97.9)	60 (38.7–78.8)	<0.0001

The optimal cutoff points were chosen based on the maximization of the sensitivity and specificity product. LDL, low density lipoprotein; HDL, high density lipoprotein; BMI, body mass index; FFM, fat-free mass; BCM, body cell mass; PhA, phase angle; TBW, total body water; AUC, area under the curve, ° Group B vs. Group C.

## Supplementary Materials

The following are available online at https://www.mdpi.com/2072-6643/12/3/742/s1, Table S1: Medical, nutritional and physical activity history, Table S2: KIDMED test to assess the Mediterranean diet adherence.
