# Supplementary material for: Impact of Vigorous-Intensity Physical Activity on Body Composition Parameters, Lipid Profile Markers, and Irisin Levels in Adolescents: A Cross-Sectional Study
Source: Nutrients. 2020 Mar 11;12(3):742. doi: 10.3390/nu12030742 (PMC7146488; doi:10.3390/nu12030742)
Supplement: Supplementary file 1 [file nutrients-12-00742-s001.zip › Supplementary Table 1.docx]

**Supplementary Table 1: Medical, Nutritional and Physical Activity History**

| General Data |
| --- |
| Date of birth (MM/DD/YYYY) ___/___/_____ |
| Sex **F M** |
| **Medical History** |
| Do you suffer any illness? **YES NO**  If yes, please specify__________________________________________________________________ |
| Are you taking any medications? **YES NO**  If yes, please specify__________________________________________________________________ |
| Are you taking dietary supplements (e.g. vitamins or minerals)? **YES NO**  If yes, please specify__________________________________________________________________ |
| Do you have any allergies? **YES NO**  If yes, please specify__________________________________________________________________ |
| Do you have any food allergies? **YES NO**  If yes, please specify__________________________________________________________________ |
| Do you have any food intolerance? **YES NO**  If yes, please specify___________________________________________________________________ |
| **Nutritional Habits** |
| Do you follow any special diet? **YES NO** |
| How many meals and snacks do you eat each day?____________________________________________ |
| What do you usually eat for snacking in the middle morning?____________________________________  _____________________________________________________________________________________ |
| What do you usually eat for snacking in the afternoon?_________________________________________  _____________________________________________________________________________________ |
| How much water do you drink?____________________________________________________________ |
| What type of beverages do you usually drink?_________________________________________________  _____________________________________________________________________________________ |
| **Physical Activity Habits** |
| Do you practice any physical activity? **YES NO**  If yes:   - What kind of sport do you practice? ____________________________________________ - Days per week______________________________________________________________ - Length of each workout (minutes)_______________________________________________ |
|  |
