# Supplementary material for: Impact of Vigorous-Intensity Physical Activity on Body Composition Parameters, Lipid Profile Markers, and Irisin Levels in Adolescents: A Cross-Sectional Study
Source: Nutrients. 2020 Mar 11;12(3):742. doi: 10.3390/nu12030742 (PMC7146488; doi:10.3390/nu12030742)
Supplement: Supplementary file 1 [file nutrients-12-00742-s001.zip › Supplementary Table 2.docx]

**Supplementary Table 2: KIDMED test to assess the Mediterranean Diet adherence**

| **N.** | **Questions** | **Scoring** |
| --- | --- | --- |
| **1.** | Takes a fruit or fruit juice every day | +1 |
| **2.** | Has a second fruit every day | +1 |
| **3.** | Has fresh or cooked vegetables regularly once a day | +1 |
| **4.** | Has fresh or cooked vegetables more than once a day | +1 |
| **5.** | Consumes fish regularly (≥ 2 times/week) | +1 |
| **6.** | Likes legumes and eats them ≥ 3times/week | +1 |
| **7.** | Consumes whole grains (pasta or rice) almost every day or ≥5 times/week) | +1 |
| **8.** | Has whole cereals or grains (bread or rusks) for breakfast | +1 |
| **9.** | Consumes nuts regularly (every day) | +1 |
| **10.** | Uses olive oil at home | +1 |
| **11.** | Has a low fat dairy product for breakfast (yogurt, milk, etc) | +1 |
| **12.** | Takes two yogurts and/or some cheese (40 g) daily | +1 |
| **13.** | Skips breakfast | -1 |
| **14.** | Has commercially baked goods or pastries for breakfast | -1 |
| **15.** | Goes >1/ week to a fast food restaurant (hamburger) | -1 |
| **16.** | Takes sweets and candy several times every day | -1 |
